# Living Alone, Internet Use, and Depressive Symptoms in Chinese Older Adults: A Longitudinal Study

**DOI:** 10.1093/geroni/igaf122.2809

**Published:** 2025-12-31

**Authors:** Wan Wen, Xiayu Chen, Kun Wang

**Affiliations:** University of Illinois Urbana-Champaign, Champaign, Illinois, United States; University of Central Florida, Urbana, Illinois, United States; University of Alabama at Birmingham, Birmingham, Alabama, United States

## Abstract

The growing trend of older adults living alone, due to rapid urbanization and family structure changes, poses unique challenges in China, where family support has traditionally been central to aging care. This shift has left many older adults with limited support from their adult children, raising concerns about increased social isolation and risk of depression. Internet use has emerged as a potential tool to mitigate these challenges by fostering social connection and engagement. However, few studies have examined the associations among internet use, living arrangement, and depressive symptoms in the Chinese context. This study examines the reciprocal relationship between Internet use and depressive symptoms and explores how this relationship varies based on living arrangements. Using two waves (2018 and 2020) of the China Health and Retirement Longitudinal Study (CHARLS), we analyzed data from 2,875 older adults (aged 60 – 95, M = 68.35 in 2018). Autoregressive cross-lagged analyses were conducted, controlling for demographic and health factors. Results revealed a unidirectional relationship among older adults living alone: more frequent Internet use predicted lower depressive symptoms two years later (B = -1.393, p = 0.003), whereas depressive symptoms did not predict Internet use. For those not living alone, no significant relationship was found. These findings suggest that older adults living alone may derive unique mental health benefits from Internet use, potentially due to its role in reducing social isolation. Policymakers and practitioners should provide more digital literacy and training programs to promote digital access among older adults, especially for those living alone.

